# The Naso-labial and lateral forehead flaps as a single stage: A case report and review of literature

**DOI:** 10.4103/0970-0358.44937

**Published:** 2008

**Authors:** I. A. Adigun, A. O. Oladele, J. K. Olabanji

**Affiliations:** Division of Plastic and Reconstructive Surgery, Department of Surgery, University of Ilorin Teaching Hospital, Ilorin, Nigeria; 1 Division of Plastic and Reconstructive Surgery Unit, Department of Surgery, Obafemi Awolowo University Teaching Hospital, Ile-Ife, Nigeria

**Keywords:** Lateral forehead flaps, nasal reconstruction, single stage

## Abstract

We present here the case of a patient with a major traumatic nasal loses who had a near-total nasal reconstruction as a single-stage procedure. A 35 year-old civil servant who was involved in a road traffic injury about two years before presentation. He sustained extensive and multiple facial injuries with complete loss of nasal cover and lining. Reconstruction was performed by using superiorly based, bilateral, nasolabial flaps to line the floor and the nasal septum, and a paramedian forehead flap for skin cover. The patient did well postoperatively and was discharged home on the 7^th^ postoperative day. If the principles concerning cover, support, and lining are adhered to, excellent functional and aesthetic results can be achieved as we have obtained in our patient.

## INTRODUCTION

The nose is a composite structure composed of the nasal skeleton, an internal lining of mucosa, and an external layer of skin. The external topography of the nose is a graceful blend of convexities, curves, and depressions that reflect the underlying shape of the nasal skeleton. The history of nasal reconstruction mirrors the history of plastic surgery. Antia and Daver as well as Mazzola and Marcus focused their historical research on the forehead flap technique for total nasal reconstruction.[1,2]

Nasal reconstruction was apparently born in Asia, most likely in India, around 3000 BC. In India, the nose was considered to be the organ of respect and reputation, nasal mutilation or amputation was therefore often used to humiliate social offenders.[3] The evolution of nasal reconstruction procedures followed three basic lines: the Indian method of a midline forehead flap; the French (Dieffenbach) method of a Lateral Cheek Flap, and the Italian method of a brachial flap. However, in 1925, Blain reviewed the various techniques available for restoration of the nose and concluded that forehead flaps worked best for major defects.[4] The nose consists of three major parts: the nasal skeleton which is the supporting structure, the nasal lining which consists of a thin layer of vascular mucosa, and the skin which proceeds inferiorly from the glabella. Nasal defects that may require reconstruction can either be due to extirpation of skin cancer, posttraumatic defects, or a congenital nasal deformity. We present here the case of a patient with a major traumatic nasal loss who had a near-total nasal reconstruction as a single-stage procedure.

## CASE REPORT

**Figure 1:**
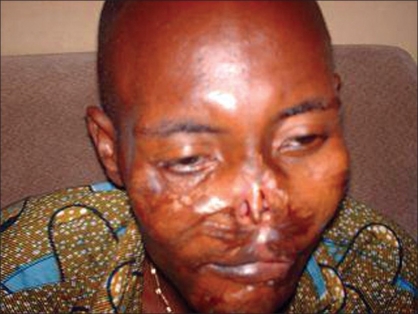
Complete loss of nasal cover and lining

**Figure 2:**
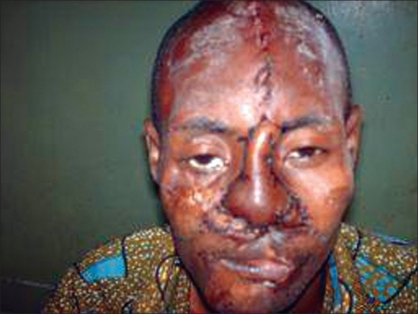
Paramedian forehead flaps for skin cover

**Figure 3:**
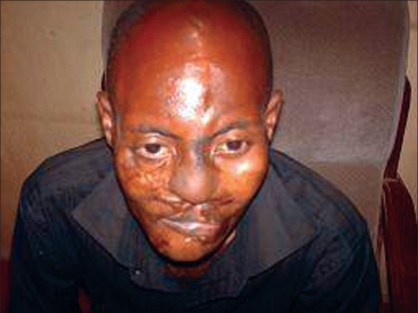
Appearance six months after surgery

A 35 year-old civil servant who was involved in a road traffic accident about two years ago. He sustained multiple injuries with an extensive facial injury which was managed in a private hospital with three operative procedures to repair the facial injury. He was discharged after ten weeks post injury. He presented to us with complete loss of nasal cover and lining as shown in Figure 1. The nasal bone and upper lateral cartilages were however intact. He had a transverse scar on the upper lip as well as a complete distortion of the normal anatomy of the upper lip. The lower lip appeared normal; he had multiple scars on the face. The patient was concerned with the loss of nasal structure and became socially withdrawn. He went out when it was absolutely necessary and covered the mid-portion of his face with a mask. He was referred to our hospital for nasal reconstruction. The patient was examined to ascertain the missing structures of the nose and besides good preoperative preparation, the mode of reconstruction and possible outcome were discussed with the patient. He was reconstructed with superiorly based, bilateral, nasolabial flaps to line the floor and the nasal septum and a paramedian forehead flap for skin cover as shown in Figure 2. The patient did well postoperatively: the stitches on the donor site of the forehead flap were removed by the 5^th^ postoperative day, and patient was discharged home on the 7^th^ postoperative day. He was seen at the follow-up clinic after four weeks and although he was satisfied with the outcome of the procedure, he did not seem to like the convexity at the root of the nose where the forehead flap was transposed. Hence, 12 weeks after the first operation, he had a refashioning of the site done under local anesthesia by a wedge excision and a linear wound closure7/1/2009 as shown in Figure 3 and was seen six months postoperatively.

## DISCUSSION

The nose is arguably the most prominent aspect of the face. It occupies a prominent place in the centre of the face, making it a structure of obvious aesthetic significance. Its reconstruction involves alteration and aesthetic details that cannot be easily hidden with clothing or apparel. In reality, recreating the nose is impossible. What nature has fabricated in a mother's womb is not reproducible, thus, the reconstructive surgeon's task can only be to fashion bits and pieces of expendable tissue into a facsimile of cover, lining, and support to give the visual impression of a normal nose. Anatomically, the nose can be divided into thirds according to its underlying skeletal structure. The proximal ⅓ of the nose rests on the nasal bones; the middle ⅓ lies over the upper lateral cartilages while the distal ⅓ or lobule includes the nasal tip with its paired alae overlying the membranous septum.[5] Before determining how to properly perform nasal reconstruction, the aesthetic and anatomic breakdown of the nose must be fully understood. When a total or subtotal reconstruction is needed, not only does the outside skin and soft tissue need to be replaced, but the nasal lining must also be reconstructed.

The cartilaginous portions of the nose, especially the lobules, are the most prominent and therefore, are most easily severed in traumatic injuries of the nose. The nasal bones, on the other hand, lie deeper and are rarely injured. We were lucky in our patient's case that both the nasal bones and the lateral cartilages were spared; this saved us the trouble of sourcing for conchal cartilages or rib cartilages and bones to be used as the skeletal elements. It is said that whenever possible, it is best to provide for nasal lining, skeletal elements, and external cover in a single operation. However, when the lining defect is very large, some surgeons feel it may be wise to stage the reconstruction and attach the proposed lining replacement to the external flap in a preliminary procedure. The cover flap can be transferred once take of the lining graft is certain. We used a superiorly based, nasolabial flap to line the nasal floor and the cartilages as a single-stage procedure. Gillies introduced bilateral nasolabial flaps turned inward to line the nasal vestibule and collumella[6], a procedure that was later refined by Millard.[7] Septal mucoperichondrial flaps which are anteriorly based, can also be used. There are, however, other multiple and complex options that can be used. Turn-in nasal flaps described by Ivy are turn-in flaps from the wound margin to replace the missing nasal lining. These flaps are hinged on the outer cicartrical edge and flipped over to span the defects.[8] A large rectangle of mucosa or a composite of mucosa and perichondrum is elevated from the septum based on the septal branch of the superior labial artery.[9] Skin grafting can be used when it is braced with cartilage to prevent graft contraction. If nasal lining is not properly performed, a contraction of the tissue can lead to an inadequate result. We were quite satisfied with the outcome of the bilateral nasolabial flap as lining that was used for our patient.

We used a paramedian forehead flap as our skin cover. This flap is the premier flap in nasal reconstruction, it can be used to replace any or all of the aesthetic subunits, and it provides excellent color and thickness match as shown in our patient. It is an axial flap based on the supraorbital and supratrochlea vessels. It has been shown that sufficient collateral blood supply from the angular arteries can sustain the flap even when the supratrochlea and supraorbital vessels are transected. The critics of the forehead flap point to the obvious forehead scarring, especially when wide flaps are designed and flap length is limited due a low hair line. Although we designed a relatively wide flap, we were able to close most parts of the defect, primarily leaving only about a 2 cm wound close to the pedicle of the flap which healed satisfactorily with no obvious scarring. At his last visit to the outpatient clinic, there was no significant hair growth on the distal part of the patient's reconstructed nose. The paramedian forehead flap has stood the test of time; it is the most useful flap that can be used for the tip, the lobule as well as subtotal and total nasal reconstruction.[10]

Other methods of skin cover include: expanded forehead flap, Gull-Winged flap, Tagliacozzi flap, and the Radial forearm free flap. Free-flap reconstruction of the nose can only be considered when the forehead is not available as a flap donor site. Free-flap tissues have poor quality in terms of color and thickness match, and the operation is riskier and more complex than elevating and transferring a forehead flap.

Our patient was seen one, three, and six months postoperatively; we tried to evaluate the alae integrity and airway patency. Although we did not augment the alae with conchal cartilages, the shape was still acceptable to our patient.

In conclusion, nasal reconstruction can be complex because it requires restoration of function with often difficult aesthetic considerations. If the principles concerning cover, support, and lining are adhered to, excellent functional and aesthetic results can be achieved. The ability to reconstruct an entire nose has opened up the possibility to patients who used to be relegated to prosthetic placement.
